# Secondary Neutropenias

**DOI:** 10.3390/biomedicines13020497

**Published:** 2025-02-17

**Authors:** Alister C. Ward

**Affiliations:** 1 School of Medicine, Deakin University, Waurn Ponds, VIC 3216, Australia; award@deakin.edu.au; 2 Institute for Mental and Physical Health and Clinical Translation (IMPACT), Deakin University, Waurn Ponds, VIC 3216, Australia

**Keywords:** cancer, chemotherapy, infection, neutropenia, neutrophil

## Abstract

Neutrophils are a critical component of immunity, particularly against bacteria and other pathogens, but also in inflammation and tissue repair. As a consequence, individuals with neutropenia, defined by a reduction in absolute neutrophil counts, exhibit a strong propensity to severe infections that typically present with muted symptoms. Neutropenias encompass a heterogeneous set of disorders, comprising primary neutropenias, in which specific genes are mutated, and the more common secondary neutropenias, which have diverse non-genetic causes. These include hematological and other cancers, involving both direct effects of the cancer itself and indirect impacts via the chemotherapeutic, biological agents and cell-based approaches used for treatment. Other significant causes of secondary neutropenias are non-chemotherapeutic drugs, autoimmune and other immune diseases, infections and nutrient deficiencies. These collectively act by impacting neutrophil production in the bone marrow and/or destruction throughout the body. This review describes the biological and clinical manifestations of secondary neutropenias, detailing their underlying causes and management, with a discussion of alternative and emerging therapeutic approaches.

## 1. Introduction

**Figure 1:**
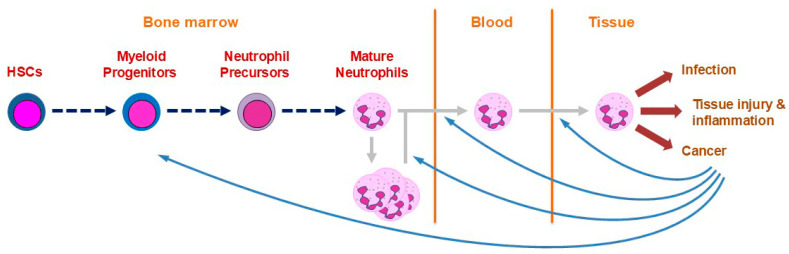
Overview of neutrophil biology. Schematic representation of the key aspects of neutrophil biology, from the production of mature neutrophils in the bone marrow from hematopoietic stem cells (HSCs) via various myeloid progenitors and neutrophil precursors. The majority of mature neutrophils remain in storage in the bone marrow with a fraction released into the blood enabling their dissemination into tissues. Here, the mature neutrophils respond to a range of insults, with these in turn able to impact neutrophil production in the bone marrow, as well as their release and migration into tissues.

Neutrophils, alternatively referred to as neutrophilic granulocytes or polymorphonuclear leukocytes (PMLs), represent a pivotal pillar of host immunity. These cells are present in large numbers but are also short-lived with increased turnover resulting from infection and other insults, which necessitates on-going production across the lifespan [1]. Neutrophils are initially generated in early embryos by transient progenitors during the primitive wave of hematopoiesis, but subsequently by hematopoietic stem cells (HSCs) in the definitive wave of hematopoiesis that occurs in the bone marrow from mid-gestation [2]. During this wave, active HSCs differentiate sequentially via multipotent progenitor (MP), common myeloid progenitor (CMP) and granulocyte-macrophage progenitor (GMP) cell populations to yield committed neutrophil precursors (NPs) with considerable proliferation occurring concurrently to expand cell numbers (Figure 1). The NP population then further differentiates to generate mature neutrophils, the majority of which are stored in the bone marrow with a small percentage entering the bloodstream where their average lifespan is 7–10 days [3].

Infections or other insults stimulate enhanced neutrophil production and release into the bloodstream [4] as well as their rapid recruitment into relevant tissues [5]. Here, neutrophils play particularly important roles in host defense against bacterial and fungal agents [1,4,5,6], but also in the context of tissue injury and acute inflammation, including their resolution [5,6,7]. These cells additionally contribute in diverse ways to both anti-tumor immunity as well as tumor pathogenesis [4,5]. Neutrophils act via a range of mechanisms to fulfill these functions, including phagocytosis [1], generation of reactive oxygen species (ROS) via cell membrane-bound nicotinamide adenine dinucleotide phosphate (NADPH) oxidase [8], release of multiple granules containing a variety of antimicrobial agents and immune mediators [5] as well as liberation of neutrophil extracellular traps (NETs) [9]. Neutrophils are also able to secrete cytokines, chemokines and other agents to regulate inflammatory responses including other immune cells [10].

An important aspect of neutrophil biology is its regulation by a range of external factors. Principal amongst these is the cytokine granulocyte colony-stimulating factor (G-CSF), also referred to as colony-stimulating factor 3 (CSF3), that plays a key role in stimulating the generation of neutrophils, as well as impacting their function [11]. G-CSF levels are normally low, but rise dramatically in response to infection, injury or inflammation [12]. G-CSF acts on HSCs and various progenitors to enhance their proliferation and lineage commitment towards neutrophils, and on NPs to stimulate their proliferation and survival/differentiation into mature neutrophils. This cytokine also enables the mobilization of mature neutrophils into circulation as well as augmenting their migration into tissues and subsequent functionality [11,13]. These properties have seen the therapeutic application of G-CSF in a range of clinical settings where neutrophil numbers are reduced [14,15,16]. The cytokine granulocyte-macrophage colony-stimulating factor (GM-CSF) acts more widely, including a key role in monocyte production and function [17], but can influence neutrophil survival [18] and chemotaxis [19]. It is employed clinically following HSCT and in conditions in which monocytes are dysfunctional or deficient [17].

A significant decrease in the number of circulating neutrophil is termed ‘neutropenia’, which can be either ‘acute’, presenting in the time-frame of hours to days, or ‘chronic’, with a time-frame of months to years [20,21]. Neutropenia can lead to increased susceptibility to infection and other pathological sequalae, the severity and frequency of which are inversely correlated with neutrophil numbers [22]. Neutropenia is classified as ‘primary’ (or ‘congenital’), where an intrinsic defect in bone marrow myeloid cells (or their precursors) is responsible [23,24], and ‘secondary’ (or ‘acquired’), where a factor extrinsic to these cells precipitates the disease [25]. This review aims to describe the different causes of secondary neutropenia, overview the management of patients with these disorders and suggest future directions.

## 2. Causes of Secondary Neutropenia

Secondary neutropenia can be precipitated by a wide variety of causes. This includes hematological and other cancers, as well as the therapies used for their treatment, but also extends to a myriad of other drugs, as well as autoimmune and other immune causes, infection by diverse pathogens, nutrient deficiencies and other causes (Table 1).

**Figure 2:**
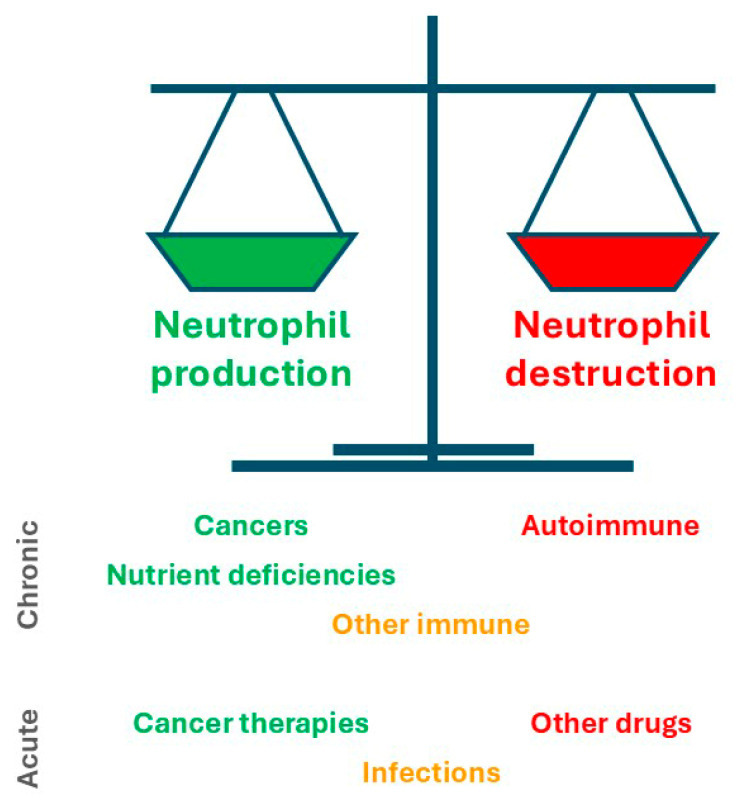
Secondary neutropenias and their impact on neutrophil production and destruction. Schematic representation of the normal balance observed in healthy individuals between neutrophil production (green) and destruction (red). The major causes of secondary neutropenia are shown indicating how they principally act to perturb this balance and over which time frame, with those that impact production in green, those that impact destruction in red and those that impact both in orange.

Like primary neutropenias, secondary neutropenias are inevitably the consequence of unbalanced neutrophil production and destruction, either acutely or chronically, with some associated with decreased production, some with increased destruction and a subset that act via both of these mechanisms [4]. This provides a useful framework to understand the various causes of secondary neutropenia (Figure 2).

### 2.1. Cancers

Cancers can directly contribute to neutropenia, with the most common mechanism being via the disruption of the bone marrow to severely impact neutrophil production by the resident HSCs. This can be mediated directly through in situ expansion of hematological neoplasms, including acute myeloid leukemia (AML), myeloproliferative neoplasms (MPNs), myelodysplastic syndrome (MDS) and multiple myeloma (MM) [26,27,28]. Alternatively, the perturbation may be caused by an infiltration of metastases derived from breast cancer, prostate cancer and other solid tumors [29], or via neoplasm-induced myelofibrosis of the bone marrow [30].

### 2.2. Cancer Therapies

#### 2.2.1. Traditional Chemotherapy

Chemotherapy is a mainstay of cancer therapy, with a wide range of agents used that kill rapidly dividing cells. While tumor cells are the target, these drugs can also severely suppress production of neutrophils by the bone marrow, particularly through their direct impact on the rapidly proliferating precursor and progenitor populations that are especially vulnerable to their cytotoxic effects [31]. Chemotherapy-induced neutropenia (CIN) is very common, with around one in six solid-tumor patients receiving traditional chemotherapy developing febrile neutropenia (FN) in the absence of prophylaxis [32]. Amongst these, lung cancer has the highest risk of FN-related hospitalization [31]. However, CIN is exacerbated in hematological malignancies compared with solid tumors [33]. CIN-associated FN principally develops in the first cycle of chemotherapy and in order to mitigate the neutropenia reductions in dosage and delays in subsequent chemotherapy cycles are necessary that adversely impacts patient outcomes [34], with the FN also impacting quality of life [35]. A multitude of chemotherapeutic agents have been implicated, including mitotic inhibitors (e.g., docetaxel, paclitaxel, vinblastine), DNA damaging agents (e.g., cisplatin, cyclophosphamide), antitumor antibiotics (e.g., doxorubicin) and anti-metabolites (e.g., hydroxyurea) [25,31,36].

#### 2.2.2. Biological Agents

Biological cancer therapies are increasingly employed especially monoclonal antibody (MAb)-based approaches to facilitate cell-type specific killing including via attached chemotherapeutic agents. Such approaches typically have a larger ‘therapeutic window’ and reduced side effects. However, a number of the agents used are also associated with neutropenia. One example is rituximab, a chimeric MAb against cluster of differentiation 20 (CD20) expressed on B cells used to provide effective treatment for non-Hodgkin lymphoma (NHL) and chronic lymphocytic leukemia (CLL) [37]. Neutropenia is especially problematic in this case since patients often also suffer from hypogammaglobulinemia amongst other immune disturbances [38]. More recently, immune checkpoint inhibitors, typically MAbs targeting critical extracellular components on T cells in particular, have revolutionized cancer therapy across multiple tumor types, notably including a range of hematological neoplasms [39]. However, some of these inhibitors have also been implicated in a range of adverse events, including neuropathy, pyrexia, gastrointestinal disturbance and leukopenia, with the latter thought to be mainly due to general immunologic enhancement [39,40,41]. This specifically includes neutropenia in the case of pembrolizumab and nivolumab that target programmed cell death protein 1 (PD-1) and ipilimumab that targets cytotoxic T-lymphocyte antigen 4 (CTLA-4) [42].

#### 2.2.3. Cell-Based Therapies

Several cell-based approaches used in oncology, and especially hematological malignancies, can result in neutropenia. These notably include HSC transplantation (HSC-T), where the pre-conditioning regime ablates neutrophil generation until the transplanted HSC can restore production [43]. Chimeric antigen receptor T cell (CAR-T) therapy has also emerged as a mediator of secondary neutropenia [44], with key risk factors being disease type and status, along with previous chemotherapy or HSCT [45]. This is mediated by ‘off-tumor’ and cytokine release-syndrome, with the latter being particularly problematic as it also induces fever in the absence of infection [45].

### 2.3. Other Drugs

A large number of non-chemotherapeutic drugs can cause neutropenia, with so-called idiosyncratic drug-induced neutropenia (IDIN) affecting around 1.6–15.4 per million per year [46,47]. Almost every drug class has been implicated, including analgesics, anticonvulsants, antimicrobials, antipsychotics, antirheumatics and antithryroids, which act by a variety of mechanisms. This can include direct toxicity but also include indirect impacts that are typically immune-mediated. Drugs can lead to hypersensitivity reactions, which can be of variable length and result in neutropenia in concert with aplastic anemia, nephritis, hepatitis and pneumonitis [46]. Alternatively, drugs can serve as haptens in a process that involves the native drug being converted to a reactive metabolite able to form adducts with proteins, especially those expressed on the neutrophil surface, which triggers the induction of an antibody response, leading to neutropenia [46]. Such immune-mediated drug-induced neutropenia has been demonstrated in the case of antithyroid drugs such as propylthiouracil, antibiotics such as penicillin, as well as aminopyrine and clozapine [46,47,48]. Biological agents employed outside of cancer therapy can also trigger enhanced immunity that impacts neutrophils, such as tocilizumab a MAb against the receptor for the cytokine interleukin 6 (IL-6R) used for the treatment of rheumatoid arthritis [49], and alemtuzumab a MAb against CD52 expressed on B and T cells in multiple sclerosis [50]. There is a genetic component for at least some IDINs, such as that mediated by clozapine [51].

### 2.4. Autoimmune and Other Immune Disorders

A variety of autoimmune neutropenias (AINs) have been described [52]. These are traditionally defined by the presence of autoantibodies directed against various epitopes found on mature neutrophils or their precursors, collectively termed human neutrophil antigens (HNAs), that typically lead to apoptosis but can also include G-CSF antibodies that block the action of this cytokine [52]. However, there is growing recognition that in addition to humoral immunity, cellular immune mechanisms should also be considered, likely involving immune initiated neutrophil destruction and suppressed production [53]. This helps explain why the levels of neutrophil autoantibodies show a poor correlation with the severity of neutropenia [52]. The majority of AIN cases emanate from an underlying autoimmune disorder, such as systemic lupus erythematosus (SLE) and Felty syndrome, and are more common in females and adults, which matches the skew seen for autoimmune diseases more generally.

Other immune-related causes include lymphoproliferative disorders, especially large granular lymphocyte (LGL) syndrome that impacts peripheral neutrophil numbers by multiple mechanisms: auto-antibody mediated apoptosis in the periphery, suppression of neutrophil production in the bone marrow, and inhibition of neutrophil egress into circulation [54]. Primary immunodeficiencies have also been implicated in secondary neutropenia that has been attributed to impacts on neutrophil survival and/or development [52], such as in the context of common variable immunodeficiency (CVID) [55]. Alternatively, maternal/fetal incompatibility for neutrophil antigens, most commonly HNA-1, has also been implicated. This occurs in 0.5–2:1000 live births with transplacental transfer of maternal immunoglobulin G (IgG) antibodies resulting in an alloimmune reaction [56]. Similar incompatibility is also observed following allogeneic HSC transplantation [57].

### 2.5. Infection

Transient neutropenia is associated with the acute and convalescent stages of a range of viral infections, but typically more pronounced with chicken pox and measles in children [58] and cytomegalovirus (CMV), Epstein–Barr virus (EBV), viral hepatitis, human immunodeficiency virus (HIV) and influenza in adults, along with coronavirus 19 (COVID-19) across the lifespan [59]. Common bacterial causes are brucellosis, rickettsia, shigellosis, tuberculosis and typhoid fever, as well as following bacterial sepsis regardless of the causative organism [58]. Other infectious agents, such as parasites and fungi, have also been implicated in neutropenia [60]. Viral-induced neutropenia is typically mild, as neutrophils redistribute from the blood to the marginating zones and into tissues where turnover occurs, but with circulating neutrophils returning to normal range within 1–2 weeks [61]. However, suppression of bone marrow neutrophil production can be observed in some adult infections typically via antibody-mediated mechanisms [62]. In the case of COVID-19, there is substantial neutrophil recruitment and activation at the site of infection [59], with extensive NETosis that appears to contribute to autoantibody production including anti-neutrophil antibodies [63]. Bacterial-induced neutropenia can be more severe and sustained with a poorer prognosis due to the depletion of bone marrow stores due to more extensive neutrophil turnover [62].

### 2.6. Nutrient Deficiencies

Nutrient deficiencies often have hematological presentations due to the relentless nature of blood cell production, including neutrophils. This includes macronutrients, as exemplified by severe protein deficit [64], various micronutrients, including both vitamin B12 and folate deficiency [65], as well as copper deficiency [66]. The resultant neutropenia typically occurs in association with other cytopenias, such as macrocytic anemia and thrombocytopenia, along with symptoms of fatigue, pallor and weight loss [67]. Somewhat underappreciated is the need for appropriate nutrition to support neutrophil recovery, which is problematic given the relatively poor nutrition found in the so-called ‘neutropenic diet’ often prescribed [68].

### 2.7. Other Causes

Many other diverse causes of secondary neutropenia have been described. These include hypersplenism, resulting in augmented clearance of damaged/aging blood cells, leading to moderate neutropenia as well as both thrombocytopenia and anemia [25]. Neutropenia can also result from maternal hypertension, which is usually self-resolving, although newborns with growth restriction may require hospitalization [69], as well as various metabolic disorders and alcohol abuse [21].

## 3. Patient Management

### 3.1. Symptoms and Diagnosis

With the exception of enhanced susceptibility to infection, including to commensal organisms, there is a general absence of clear symptomatology for neutropenias, including secondary forms [20,70,71]. Fever is often the sole indication of such infections, since the paucity of neutrophils results in typical infection-mediated inflammation and other sequalae such as mouth ulcers being muted or altogether absent [20,21].

The risk of severe infection is dependent on both the underlying pathology and duration of neutropenia, with risk increased in those secondary neutropenias associated with decreased neutrophil production and those that are chronic [67]. Gram-negative bacteria represent the leading cause of immediate infections, especially Enterobacteriaceae and *Pseudomonas aeruginosa*, with common Gram-positive causes being *Staphylococcus* spp. and *Enterococcus* spp. [72,73]. Fungal agents tend to infect after sustained neutropenia, with *Aspergillus* spp. and *Candida* spp. being the most common [73,74], and being particularly problematic in patients with hematological malignancy [75].

Febrile patients are assessed for infection principally by culturing. Typically, blood and urine samples are cultured for bacterial and fungal growth, mouth ulcers for herpes virus, and potentially stool and sputum cultures for enteric and pulmonary bacteria, respectively, depending on the patient’s clinical signs [20,21]. These investigations can be supplemented with chest X-rays, although computed tomography (CT) scans offer greater sensitivity and are also applicable to the paranasal sinuses and abdomen if indicated by the patient’s symptoms [76].

Diagnostic confirmation of secondary neutropenia requires a complete blood count. However, this is not necessarily straightforward, since neutrophil numbers are notoriously variable in comparison to other blood cell parameters, with infection status, levels of activity, medication and various health conditions all known to contribute to short-term fluctuations [20]. The typical cut-off point for neutropenia in adults is 1.5 × 10^9^/L, with 1.0–1.5 × 10^9^/L classified as ‘mild’, 0.5–1.0 × 10^9^/L as ‘moderate’ and <0.5 × 10^9^/L as ‘severe’ [20,21]. However, these threshold levels are strongly influenced by age and ethnicity, being higher for neonates and infants, and lower for certain ethnic groups such as African Americans [77]. Histological analysis may also give insight into the cause; for example, the presence of blast cells, dysplastic cells or hypersegmented neutrophils in AML, MDS or nutrient deficiency, respectively [71].

To further investigate the underlying causes of secondary neutropenia, a full patient history is essential, particularly regarding other illnesses, medications and family history [70]. Physical examination is employed to assess potential lymphadenopathy, splenomegaly and lesions of the mouth and skin [21]. In the absence of an obvious cause, bone marrow examination may be utilized to evaluate whether production or destruction is affected, with the potential to also identify specific hematological disease causes [20]. If immune-mediated causes are suspected anti-neutrophil antibodies can be tested [52], or copper, folate and vitamin B12 levels in the case of nutritional deficiency [67].

### 3.2. Treatment

A number of tools have been developed to assess risk in neutropenia patients, notably including the Multinational Association for Supportive Care in Cancer (MASCC) [78] and the Critical Index of Stable Febrile Neutropenia (CISNE) [79]. These have been incorporated into several clinical guidelines that inform treatment with proven effectiveness [80,81,82]. In the majority of secondary neutropenia cases, the most critical necessity is the urgent treatment of suspected infections. Febrile patients are typically treated with high-dose, broad-spectrum antibiotics delivered intravenously, with the specific agents informed by pathogen identification and sensitivity testing performed on the patient [83]. Anti-fungal therapies are indicated if the fever remains sustained [84]. Anti-microbial agents, such as amphotericin B for invasive aspergillosis [85], and antibody-based therapy [84] can be given prophylactically to afebrile neutropenic patients. Neutropenic diets that aim to reduce the risk of infection via the gut are often recommended [86]. Cytokine therapy has also been used to treat patients with secondary neutropenia. This includes G-CSF, which can effectively restore neutrophil numbers, although its effectiveness in improving patient outcomes is controversial [25]. GM-CSF has also been shown to shorten neutrophil recovery following chemotherapy or radiation exposure [87].

More tailored approaches are employed depending on the underlying cause. For patients with secondary neutropenia due to cancer, treatment of the cancer necessarily takes precedent, which may involve cancer therapies including chemotherapy and HSCT that can worsen the neutropenia [88]. However, G-CSF has proven to be effective as a prophylactic agent against febrile neutropenia prior to intensive chemotherapy or HSCT [14,89]. Moreover, GM-CSF has proven to be an effective adjuvant during checkpoint inhibitor immunotherapy, where it can abrogate the immune-related adverse events, while also enhancing its anti-cancer impacts [17]. For cases of IDIN, the most important action is drug cessation with recovery occurring after approximately 9 days [56,71]. However, this is not always straightforward for patients with multiple medications, or those where there is no effective alternative medication available, such as the case with certain psychiatric drugs [46]. Nutrient deficiencies are typically easy to overcome through dietary interventions, while secondary neutropenia induced by infection will generally resolve once the infection is cleared [21].

## 4. Future Directions

There remain many areas surrounding secondary neutropenia and its management that can be improved and/or would benefit from further research and development. There is a need for a more personalized approach to care. This can be facilitated, for example, by the development and use of standardized reference ranges across racial groups to better diagnose clinically relevant neutropenia [90] and application of patient-specific risk assessments [91], including the use of machine learning approaches for the analysis of patient data that have been used to identify those at high risk of developing CIN [92].

The use of broad-spectrum antibiotics in FN patients is problematic from several perspectives, such that its cost–benefit remains contentious. Its effectiveness is undermined by antibiotic-resistance [93], altered pharmacokinetics caused by the neutropenia [94], and in cases when the FN does not have an infectious cause [43], such as in cytokine release-syndrome common in CAR-T patients [45]. Furthermore, use of antimicrobial agents is associated with an increased risk of *Clostridioides difficile* infection, the emergence of multi-drug-resistant organisms and microbiome dysbiosis [43]. Therefore, early de-escalation or discontinuation of antibiotics is recommended to minimize adverse sequalae [83]. Other mitigation strategies include screening for resistant bacteria and rapid diagnostic assays [93,95]. A recent innovation has been the application of metagenomics next-generation sequencing (NGS), which has been shown to provide a more comprehensive pathogen identification in an unbiased, culture-independent manner to allow for timely optimization of anti-infective therapies [96,97]. However, issues such as cost, availability, standardization and interpretation of results need to be overcome for it to be widely used [98]. Preventative non-pharmacological approaches are also being employed, such as use of high-efficiency particulate air (HEPA) filters and cessation of smoking [99]. There has also been a shift away from dietary limitation to a focus on appropriate food storage, preparation and cooking to mitigate infection via the gut [86].

The use of G-CSF has also proven to be challenging. This includes from a pharmacologic perspective, with a need for on-going injections and sub-optimal neutrophil recovery often seen [100], while debilitating side-effects are relatively common, with bone pain resulting from bone marrow expansion, acute inflammation and enhanced nerve fiber sensitivity being the primary adverse event [101]. However, G-CSF treatment can also be problematic from a pathophysiologic perspective [102] due to the pro-tumorigenic roles reported for neutrophils in advanced cancer [103] and their pro-inflammatory role in autoimmune disease [104]. Exciting new approaches are becoming available that address some of these issues. For example, long-lasting versions of G-CSF are being applied in non-myeloid malignant tumors to optimize neutrophil recovery, including two recombinant Fc receptor/G-CSF fusion proteins, efbemalenograstim alfa [105] and eflapegrastin-xnst [100], with on-body injectors being utilized as an alternative strategy [106]. Meanwhile, GM-CSF, corticosteroids and intravenous immunoglobulin are being employed in patients in which G-CSF therapy is contraindicative [17,102].

A number of alternative treatments are being developed for application in CIN. These include the cyclin-dependent kinase 4/6 (CDK4/6) inhibitor trilaciclib [107] that induces a transient and reversible arrest of hematopoietic stem and progenitor cells, thereby protecting them from the neutropenic impacts of chemotherapeutic agents that remain effective against the tumor cells [108]. Another example is plinabulin, which binds to a pocket of β-tubulin to prevent microtubule polymerization. This agent serves to increase myeloid progenitor cells to compensate for the impacts of chemotherapy agents [109], but also exerts various anti-tumor effects, such as promoting the so-called ‘M1-like’ pro-inflammatory macrophages [110], inhibiting angiogenesis and inducing apoptosis [111]. Both agents have shown efficacy in clinical trials, without the bone pain and need for on-going administration that occurs with G-CSF therapy, with nausea and fatigue being common adverse events [31].

## 5. Conclusions

Secondary neutropenias represent common disorders, occurring as a consequence of a variety of causes. Hematological neoplasms and solid tumor metastases, as well as the chemical and biological agents used for cancer therapy can directly impact neutrophil production by the bone marrow. In addition, non-chemotherapeutic drugs, autoimmune and other disorders, infection and nutrient deficiency can result in neutropenia through impacts on neutrophil production and/or destruction. The major clinical consequence of secondary neutropenia is a greatly enhanced susceptibility to infection that is typically manifested as a somewhat muted fever. Traditional treatments have focused on broad-spectrum antibiotics, neutropenic diets and prophylactic G-CSF therapy, although their use is not always well supported by empirical data. However, this is shifting to a more patient-centered approach, with tailored use of anti-infectives and novel biological agents in concert with other sophisticated strategies to mitigate the impacts on the patient.

## Figures and Tables

**Table 1:** Major causes of secondary neutropenia.

Cause	Type	Examples
Cancers	hematological	AML, MDS, MM, MPN
	solid tumor metastases	breast, pancreatic
Cancer therapies	chemotherapeutic agents	cisplatin, cyclophosphamide, docetaxel, doxorubicin, hydroxyurea, paclitaxel, vinblastine
	biological agents	ipilimumab, nivolumab, pembrolizumab, rituximab
	cell-based therapies	CAR-T therapy, HSCT
Other drugs	analgesics/NSAIDs	aspirin, diclofenac, ibuprofen, tenoxicam
	anticonvulsants	carbamazepine, phenytoin, valproate
	antimicrobials	acyclovir, chloroquine, levamisole, penicillins, tetracycline, trimethoprim-sulfamethoxazol
	antipsychotics	amoxapine, clozapine, diazepam, olanzapine, phenothiazines
	antirheumatics	gold, levamisole
	antithyroids	carbimazole, methimazole, propylthiouracil
	non-cancer biologicals	tocizulimab
	other	aminopyrine, deferiprone, digoxin, glucocorticoids, octreotide, ramipril
Autoimmune and other immune disorders	autoimmune disorders	Felty’s syndrome, SLE
	lymphoproliferative disorders	LGL
	immunodeficiencies	CVID
	incompatibility	maternal/fetal, HSCT
Infection	viral	chickenpox, CMV, COVID-19, EBV, HIV, influenza, measles, viral hepatitis
	bacterial	brucellosis, rickettsia, sepsis, shigellosis, typhoid fever, tuberculosis
	other	malaria
Nutrient deficiencies	macronutrients	protein
	micronutrients	copper, folate, vitamin B12
Other causes		hypersplenism, maternal hypertension

Abbreviations: AML—acute myeloid leukemia; CAR-T—chimeric antigen receptor T cell; CMV—cytomegalovirus; COVID—coronavirus disease; CVID—common variable immune deficiency; EBV—Epstein–Barr virus; HIV—human immunodeficiency virus; HSCT—hematopoietic stem cell transplantation; LGL—large granular leukemia; MDS—myelodysplastic neoplasm; MM—multiple myeloma; MPN—myeloproliferative neoplasm; SLE—systemic lupus erythematosus.

## Data Availability

No new data were created or analyzed in this study. Data sharing is not applicable to this article.
